# A Long-Term High-Fat/High-Sucrose Diet Promotes Kidney Lipid Deposition and Causes Apoptosis and Glomerular Hypertrophy in Bama Minipigs

**DOI:** 10.1371/journal.pone.0142884

**Published:** 2015-11-16

**Authors:** Li Li, Zhanzhao Zhao, Jihan Xia, Leilei Xin, Yaoxing Chen, Shulin Yang, Kui Li

**Affiliations:** 1 Key Laboratory of Farm Animal Genetic Resources and Germplasm, Innovation of Ministry of Agriculture, Institute of Animal Science, Chinese Academy of Agricultural Sciences, Beijing, China; 2 College of Veterinary Medicine, China Agricultural University, Beijing, China; 3 Agricultural Genomes Institute at Shenzhen, CAAS, Shenzhen, P.R. China; University of Louisville, UNITED STATES

## Abstract

Metabolic syndrome can induce chronic renal injury in humans. In the present study, Bama minipigs were fed a high-fat/high-sucrose diet (HFHSD) for 23 months, which caused them to develop the pathological characteristics of metabolic syndrome, including obesity, hyperinsulinemia, and hyperlipidemia, and resulted in kidney tissue damage. In the HFHSD group, the ratio of the glomus areas to the glomerulus area and the glomerular density inside the renal cortex both decreased. Lipid deposition in the renal tubules was detected in the HFHSD group, and up-regulated expression levels of SREBP-1, FABP3 and LEPR promoted lipid deposition. The decreased levels of SOD, T-AOC and GSH-PX indicated that the antioxidant capacity of the renal tissues was diminished in the HFHSD group compared with MDA, which increased. The renal tissue in the HFHSD group exhibited clear signs of inflammation as well as significantly elevated expression of key genes associated with inflammation, including tumor necrosis factor-α (TNF-α) and macrophage migration inhibitory factor (MIF), compared with the control group. The tubular epithelial cells in the HFHSD group displayed significantly greater numbers of apoptotic cells, and the expression of proliferating cell nuclear antigen (PCNA) in the renal tubules decreased. Caspase-3 expression increased significantly, and the transcription factor nuclear factor κB (NF-κB) was activated and translocated into the nucleus. In conclusion, long-term HFHSDs cause metabolic syndrome and chronic renal tissue injury in Bama minipigs. These findings provide a foundation for further studies investigating metabolic syndrome and nephropathy.

## Introduction

Metabolic syndrome is an emerging chronic disease in humans that includes obesity, high blood sugar, high blood pressure, high cholesterol, and insulin resistance [1–3]. Patients with metabolic syndrome display chronic renal tissue injury that leads to chronic renal disease and, in severe cases, end-stage renal failure [4–7]. Chronic renal disease severely endangers human health, not only by causing renal tissue injury but also by contributing to cardiovascular disease [8–10].

Metabolic syndrome includes obesity and dyslipidemia. Increased lipid levels lead to abnormally elevated blood lipid concentrations. As a blood filtration organ, the kidney also becomes enriched with lipids. As a consequence of other mechanisms, renal tissue lesions occur when the lipid reabsorption capacity of the renal tubules is reached [11, 12]. Renal tissue lesions occur during two major pathological stages: hypertrophy (early stage) and sclerosis (late stage). Early stage renal tissue hypertrophy is associated with glomerular cell proliferation. Glomerulosclerosis and tubulointerstitial fibrosis occur in the late stage. To prevent chronic renal tissue injury caused by metabolic syndrome, it is necessary to better understand the association between metabolic syndrome and renal tissue injury, as well as its underlying mechanism. Obesity and high blood sugar levels and blood lipid levels, similar to those reported for human metabolic syndrome, have been reported in mice and rats fed high-fat diets, together with corresponding renal injury [13–17]. However, Dissard et al. conducted a long-term study using high-fat and high-fructose diets in mice and showed that mice were not an ideal model to assess renal diseases because of dissimilarities in the types of urinary peptides present between mice and humans [18].

Minipigs are omnivorous animals [19]. There are many similarities between miniature pigs and humans, including their preferred normal diet, sugar and lipid metabolism [20], anatomy, physiology, and organ morphology. In this study, Bama minipigs were fed a high-fat/high-sucrose diet (HFHSD) for 23 months and developed the characteristics of metabolic syndrome. The pathology of the liver [21, 22], heart [23] and pancreas [24] were analyzed in these animals. Based on an analysis of renal tissue pathology and key molecular characteristics, we demonstrated that Bama minipigs develop renal tissue injury as a consequence of metabolic syndrome.

## Materials and Methods

### Experimental animals

A total of 12 six-month-old Bama minipigs were randomly divided into two groups: six animals in the control diet (CD) group (143, 147, 157, 159, 161, 163) and six animals in the experimental HFHSD group (120, 12, 1368, 140, 144, 146) [21–24]. The CD group was fed basic feed, and the experimental group was fed a HFHSD mixture of 53% basic feed, 37% sucrose, and 10% lard (Table 1) [25, 26]. The animals were fed twice daily, and the amount of food provided was 3% of the body weight of the animal. A feeding cycle of 23 months was followed. The animals were housed at the animal experimental base in the Agriculture Institute of Animal Science of the Chinese Academy of Agricultural Sciences. The animals were allowed access to water *ad libitum*, and the room temperature was 20–25°C. The animals were bred using conventional procedures. The animals used in this study received humane care according to the criteria outlined in the “Guide for the Care and Use of Laboratory Animals, ISA, CAAS”, and all of the procedures were approved by the Animal Care and Use Committee of the Germplasm Resource Center of Chinese Experimental Minipigs.

**Table 1:** Nutrition content of the feed:

Components		Control Diet	HFHSD
Corn	%	71.55	37.92
Soybean meal	%	14.32	7.58
Wheat bran	%	4.14	2.19
Colza meal	%	5	2.65
Stone powder	%	1	0.53
Salt	%	0.38	0.20
Fish powder	%	2	1.06
Phosphate calcium	%	1.02	0.54
Trace elements	%	0.075	0.039
Multivitamin	%	0.015	0.008
Premix feed	%	0.50	0.26
Pork lard	%	0	10
Sucrose	%	0	37
Crude protein	%	17.8	8.7
Crude fat	g/kg	34	150
Crude fiber	%	3.7	2.7
Moisture content	%	7.6	4.5
Reducing sugar	g/100 g	0.68	2.6
Amylum	g/100 g	59.9	41.2
Ferrum	mg/kg	230	130
Cholesterin	mg/100 g	15.8	8.6

### Analysis of blood biochemistry

After overnight fasting, the body weight of the animals was determined. Whole blood was collected and centrifuged at 3500 rpm and 4°C for 10 min to separate the serum. The serum glucose concentration was measured using the immobilized glucose oxidase method. Serum insulin was measured using the radioimmunoassay method. The homeostasis model assessment (HOMA-IR = fasting insulin * fasting glucose /22.5) was used to evaluate insulin resistance [27–29]. Serum triglycerides (TGs), total cholesterol (TC), low-density lipoprotein cholesterol (LDL-C), high-density lipoprotein cholesterol (HDL-C), leptin, serum creatinine, serum urea nitrogen and serum albumin were assessed using an H7600-020 automatic analyzer (Hitachi, Japan).

### Hematoxylin-eosin and Oil Red O staining

Tissues from the same locations in the kidneys of the minipigs were collected (including the cortex and medulla) and fixed in 4% paraformaldehyde and 85% ethanol for conventional paraffin sectioning at a thickness of 6 μm. The structure of the renal tissue was observed by hematoxylin-eosin (HE) and periodic acid–Schiff (PAS) staining. The thicknesses of the cortices and medullas were measured to calculate the cortex/medulla ratio using an H-7500 (Japan) electron microscope and Image-Pro Plus (IPP; Media Cybernetics Corporation, Maryland, USA). Under the 40X eyepiece of an H-7500 (Japan) electron microscope, five random fields were selected to calculate the areas of the glomeruli and the tubular unit. Frozen sections were stained with Oil Red O to examine the accumulation of lipids. The sections were observed using an H-7500 electron microscope.

### Analysis of antioxidant capacity

Renal tissues were collected and analyzed using a superoxide dismutase (SOD) detection kit (Sigma, St. Louis, MO, USA) to measure SOD activity. A glutathione peroxidase (GSH-PX) detection kit (Sigma, St. Louis, MO, USA) was used to measure GSH-PX activity, and a total antioxidant capacity (T-AOC) detection kit (Sigma, St. Louis, MO, USA) was used to determine T-AOC. A lipid peroxidation (malondialdehyde, MDA) kit (Sigma, St. Louis, MO, USA) was used to assay lipid peroxidation (MDA).

### Immunohistochemistry

The sections were deparaffinized with xylene and then rehydrated. After antigen retrieval, the sections were incubated in 3% H_2_O_2_ for 30 min. After blocking in serum, mouse anti-proliferating cell nuclear antigen (PCNA) monoclonal (Sigma, St. Louis, MO, USA) and rabbit anti-caspase-3 polyclonal (Sigma, St. Louis, MO, USA) antibodies were added to the sections and incubated at 4°C overnight. After washing with PBS, biotinylated horse anti-mouse IgG (Beijing CW Biotech, Beijing, China) and biotinylated goat anti-rabbit IgG (Beijing CW Biotech, Beijing, China) were added to the sections and incubated at room temperature for 2 h. Next, the sections were washed with PBS and incubated with horseradish peroxidase-labeled streptavidin (Sigma, St. Louis, MO, USA) at room temperature for 2 h. The samples were developed using DAB and counterstained with hematoxylin. Positive cells displayed a brown-yellow coloration. The negative control sections were incubated in PBS instead of the primary antibody. The positive expression rates of PCNA, caspase-3, and NF-κB were calculated. Five fields in each section were randomly selected to calculate the total number of cells and the number of positive cells in the renal tubules. The data are presented as percentages.

### Real-time quantitative PCR

TNF-α, IL-6, and MIF are key inflammatory genes in pathological kidney injury [30–32]. PCNA and VEGFA are involved in the thickening of the capsular basement membrane [33, 34], and SREBP-1, LEPR, and FABP3 are involved in lipid deposition in the kidney [34].

The expression levels of these genes in the six minipigs in the CD (143, 147, 157, 159, 161, and 163) and HFHSD (120, 126, 138, 140, 144, and 146) groups were evaluated by qRT-PCR. Total RNA from pig kidney tissue was extracted using TRIzol (Invitrogen, Beijing, China) and treated with RNase-free DNase. The PCR primers used for the analysis are listed in Table 2. First-strand cDNA synthesis was performed using MMLV reverse transcriptase (Promega, Madison, USA) according to the manufacturer’s instructions. The qRT-PCR reaction was conducted using a 7500 real-time PCR system (Applied Biosystems) with the following parameters: denaturation for 2 min at 95°C, followed by 30 s at 60°C and 30 s at 72°C. The relative gene expression levels were calculated using the threshold cycle (Ct value). The 2−ΔΔCT method was used to analyze the expression levels of the target genes. Three biological replicates were analyzed to evaluate statistical significance. The data are presented as the mean values ± standard deviation.

**Table 2:** qRT-PCR primers used in the present study.

Gene	Forward primer (5’-3’)	Reverse primer (5’-3’)
TNF-α	5'TCGACTCAGTGCCGAGAT3'	5'ATAATAAAGGGATGGACAG3'
IL-6	5'GGATGCTTCCAATCTGGGTT3'	5'GTGGCTTTGTCTGGATTCTTC3'
MIF	5' CCGTGCGCCCTTTGCAGTCT 3'	5' GCCGTTCCAGCCCACATT 3'
PCNA	5' CGTGAACCTCACCAGCAT 3'	5' ATTTGGAGCTTCAAACACT 3'
VEGFA	5' CGAGACCCTGGTGGACAT 3'	5' TCCAGACCTTCGTCGTTG 3'
SREBP-1	5' ACGCCGCCTCCTTCCACCAT 3'	5' AAGGCAGGCACCGACGGGTA 3'
LEPR	5' TCATTGTGCAGCGTGAGA 3'	5' ATTGAATTGACGGCCAGA 3'
FABP3	5' ATGACCAAGCCTACCACA 3'	5' TCATCAAACTCCACTCCC 3'

### Statistical analysis

The data were analyzed using SPSS17.0 statistical software. Significant differences between groups were determined using the independent sample t-test. The results are presented as the mean values ± standard deviation (X ± SD). P < 0.05 was considered statistically significant.

## Results

### Minipigs fed long-term high-fat/high-sucrose diets develop metabolic syndrome

The minipigs in the HFHSD group developed obesity, hyperinsulinemia, increased blood lipid concentrations, and renal inflammatory responses after 23 months of feeding (Table 3). Compared with the CD group, the HFHSD group demonstrated a 2.73-fold increase in body weight (P < 0.001). The fasting blood glucose levels did not differ significantly between the HFHSD group and the CD group (P > 0.05). The level of fasting insulin in the HFHSD group was 4.96 times higher than that in the CD group (P = 0.001), and the HOMA-IR was 3.77 times higher in the HFHSD group compared with the CD group (P = 0.008). These results indicate that the minipigs in the HFHSD group had hyperinsulinemia. In addition, the total blood cholesterol was 2.89 times higher in the HFHSD group than in the CD group (HDL-C was 3.59 times higher, LDL-C was 2.12 times higher, TGs were 3.91 times higher, and leptin was 1.55 times higher in the HFHSD compared with the CD group).

**Table 3:** Characteristics of the minipigs in the HFHSD and CD groups.

	HFHSD	Control	P value
Body weight (kg)	140.28 ± 8.52	51.30 ± 5.85	< 0.001*
Blood glucose (mmol/L)	4.99 ± 1.11	6.62 ± 0.85	0.061
Insulin (μIU/L)	28.32 ± 7.84	5.71 ± 0.39	0.001*
HOMA-IR (fasting insulin * fasting glucose /22.5)	5.58 ±1.38	1.48 ±0.16	0.009*
Serological indicators			
TC (μmol/L)	3.41 ± 0.29	1.18 ± 0.38	0.002*
HDL-C (μmol/L)	0.97 ± 0.52	0.27 ± 0.07	0.038*
LDL-C (μmol/L)	1.32 ± 0.17	0.62 ± 0.17	0.004*
TG (μmol/L)	1.72 ± 0.32	0.44 ± 0.19	< 0.001*
Leptin (ng/ml)	1.57 ± 0.74	1.01 ± 0.23	0.037*
Kidney morphology			
Glomerular density (n/50,000 μm2)	1.76 ± 0.27	2.34 ± 0.42	0.006*
Glomus area (μm2)	16214.86 ± 5806.87	8853.90 ± 2475.30	< 0.001*
Glomerular area (μm2)	26911.72 ± 9492.64	14088.41 ± 3801.94	< 0.001*
Glomus area/glomerular area (%)	60.26 ± 5.58	62.73 ± 4.97	0.047*
Cortex thickness/medullary thickness (%)	2.25 ± 0.32	2.49 ± 0.77	0.436
Kidney function			
Serum albumin (g/L)	38.22 ± 2.07	39.17 ± 2.45	0.558
Serum urea nitrogen (μmol/L)	1.54 ± 0.23	3.12 ± 0.52	< 0.001*
Serum creatinine (μmol/L)	117.42 ± 14.38	176.67 ± 6.33	< 0.001*

The values are the mean ± standard error.

* P<0.05 compared with the control group.

### Long-term high-fat/high-sucrose diets induce glomerular hypertrophy

**Fig 1:**
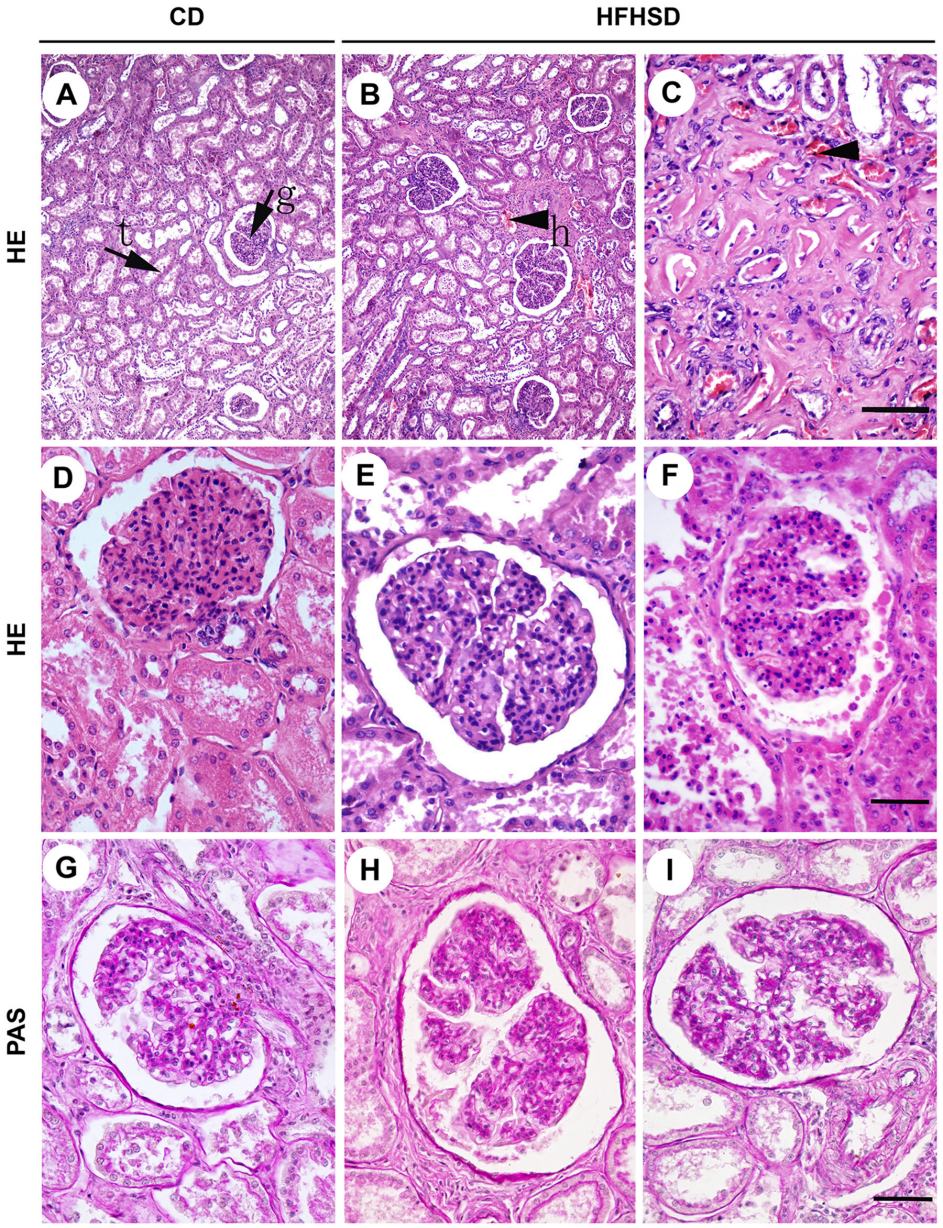
Comparison of HE and PAS Staining of the Renal Tissues in the HFHSD and CD Groups. A, B, C. HE staining; Bar = 200 μm. D, E, F. HE staining; Bar = 50 μm. G, H, I. PAS staining Bar = 50 μm. g: glomerular; t: kidney tubules; h: hyperemia. The HFHSD group displayed enlarged glomeruli with deep PAS staining and blood vessel congestion. CD = control diet; HFHSD = high-fat/high-sucrose diet.

**Fig 2:**
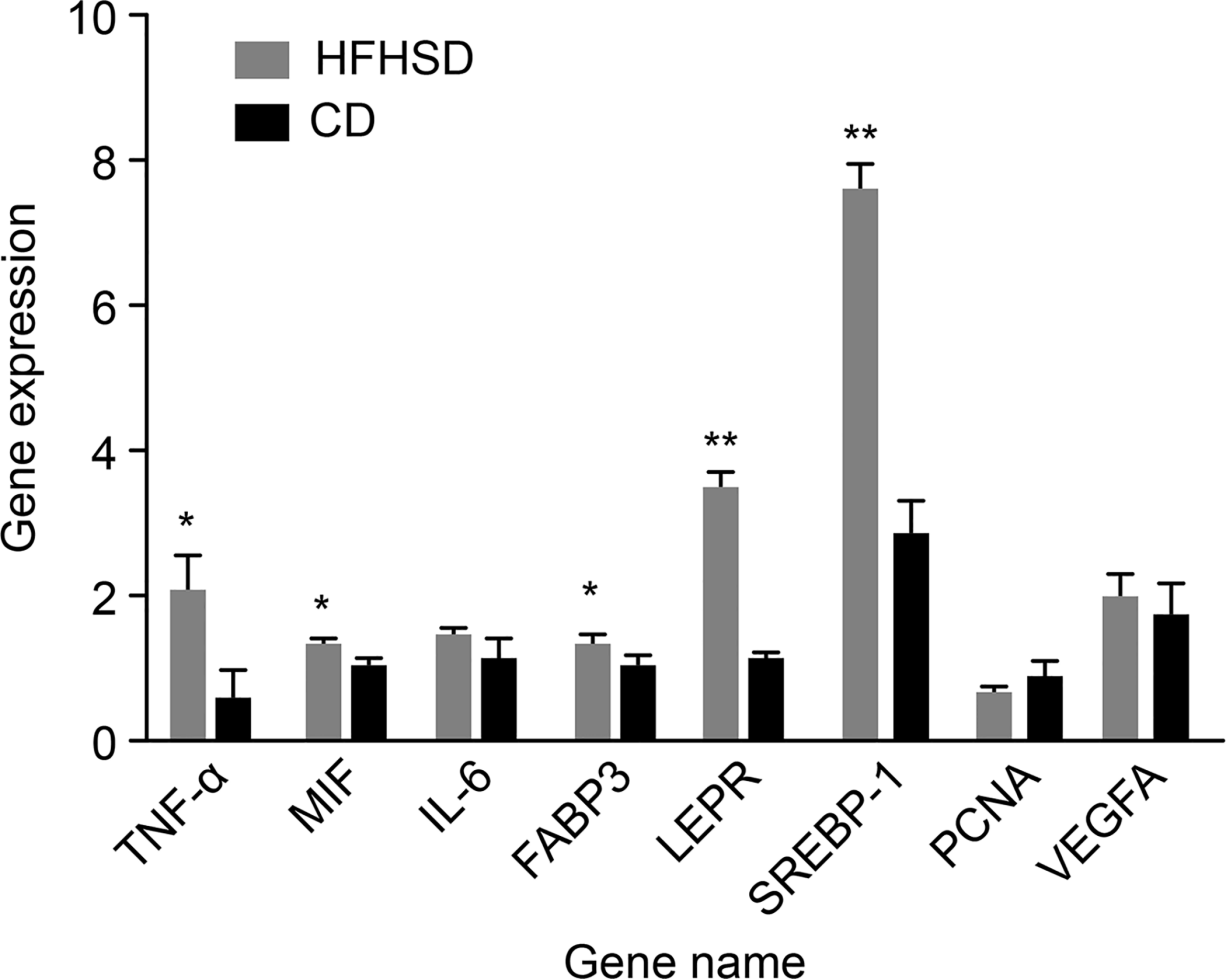
Gene Expression Levels Assessed by qRT-PCR. Evaluation of the expression levels of metabolic syndrome-related genes by qRT-PCR. Notes: *P < 0.05 HFHSD vs. CD; **P < 0.01 HFHSD vs. CD.

HE staining (Fig 1) demonstrated that the glomerular structure was intact in the CD group, and the boundary between the renal capsule and the glomus was clear. The tubular epithelial cells were clearly defined, well arranged, and similar in size. The glomerular volume was significantly increased in the HFHSD group, with mesangial matrix hyperplasia (positive PAS staining), glomerular swelling, an irregular morphology of some glomeruli, lobulated capillary loops, and a significantly increased renal capsular space. However, there were no significant differences in PCNA and VEGFA expression between the two groups (Fig 2). The tubular epithelial cells were disordered in the HFHSD group. The interstitial blood vessels were dilated, with red blood cell exudation (arrow) and blood vessel congestion in both the cortex and the medulla. Some of the renal epithelial cells displayed swelling and vacuolation, while some of the renal tubule cells had ruptured on their luminal surfaces (Fig 1). The HFHSD and CD groups had a glomus area of 16214.86 ± 5806.87 μm^2^ and 8853.903 ± 2475.30 μm^2^ (P < 0.001) and a glomerular area of 26911.72 ± 9492.64 μm^2^ and 14088.41 ± 3801.94 μm^2^ (P < 0.001), respectively. In addition, the ratio of the glomus to the glomerulus area decreased 3.94% (P = 0.047). The glomerular density inside the renal cortex decreased 24.79% (P = 0.006) in the HFHSD group compared with the CD group; however, the difference in medullary thickness between the two groups was not significant (P = 0.436). The serum albumin in HFHSD and CD groups was 38.22 ± 2.07 and 39.17 ± 2.45 (P = 0.558), respectively. In CD group the serum urea nitrogen (P<0.001) was 3.12 ± 0.52, the serum creatinine (P<0.001) was 176.67 ± 6.32, and in HFHSD group were 1.54 ± 0.23 and 117.42 ± 14.38 (Table 3), respectively. These indicators are relevant of the renal function.

**Fig 3:**
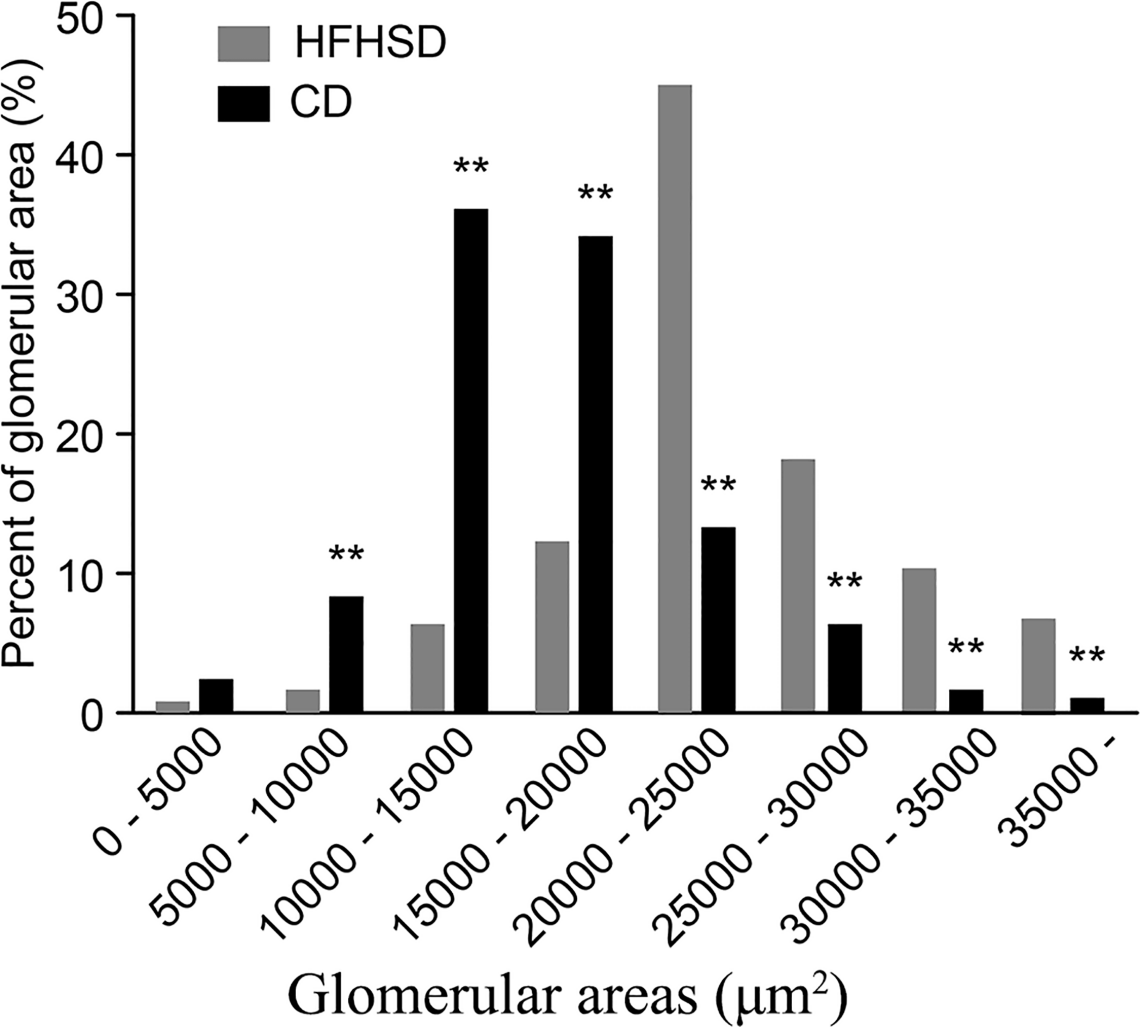
Effects of High-Fat/High-Sucrose Diets on the Glomerular Area in Bama Minipigs. Effect of HFHSD on the glomerular area in Bama minipigs. Notes: **P < 0.01 HFHSD vs. CD.

The statistical analysis of the glomerular areas revealed that the glomerular areas in the two groups followed a normal distribution. In the CD group, the peak value occurred between 1.0×10^4^ and 1.5×10^4^ μm^2^, while the peak value in the HFHSD group occurred between 2.0×10^4^ and 2.5×10^4^ μm^2^. The peak value in the HFHSD group was 27.16% higher than that in the CD group. In addition, the glomerular areas in the HFHSD group that were larger than 20,000 μm^2^ were 293.20% larger than those in the CD group, while the glomerular areas in the CD group that were smaller than 20,000 μm^2^ were 324.40% smaller than those in the HFHSD group (Fig 3).

### HFHSDs diets promote lipid deposition and inflammatory responses in the kidney

**Fig 4:**
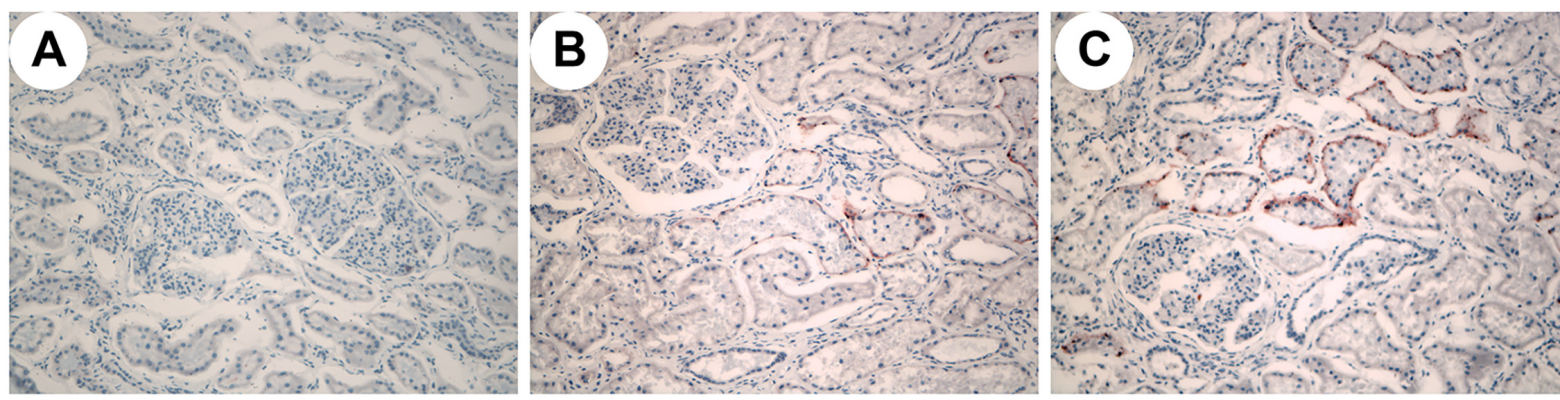
Oil Red O Staining of Renal Tissues in Bama Minipigs in the HFHSD and CD Groups. A. (200x) CD group. B,C (200x) HFHSD group. No lipid deposition was observed in the CD group. Obvious lipid deposition was observed in the renal tubules in the HFHSD group.

**Fig 5:**
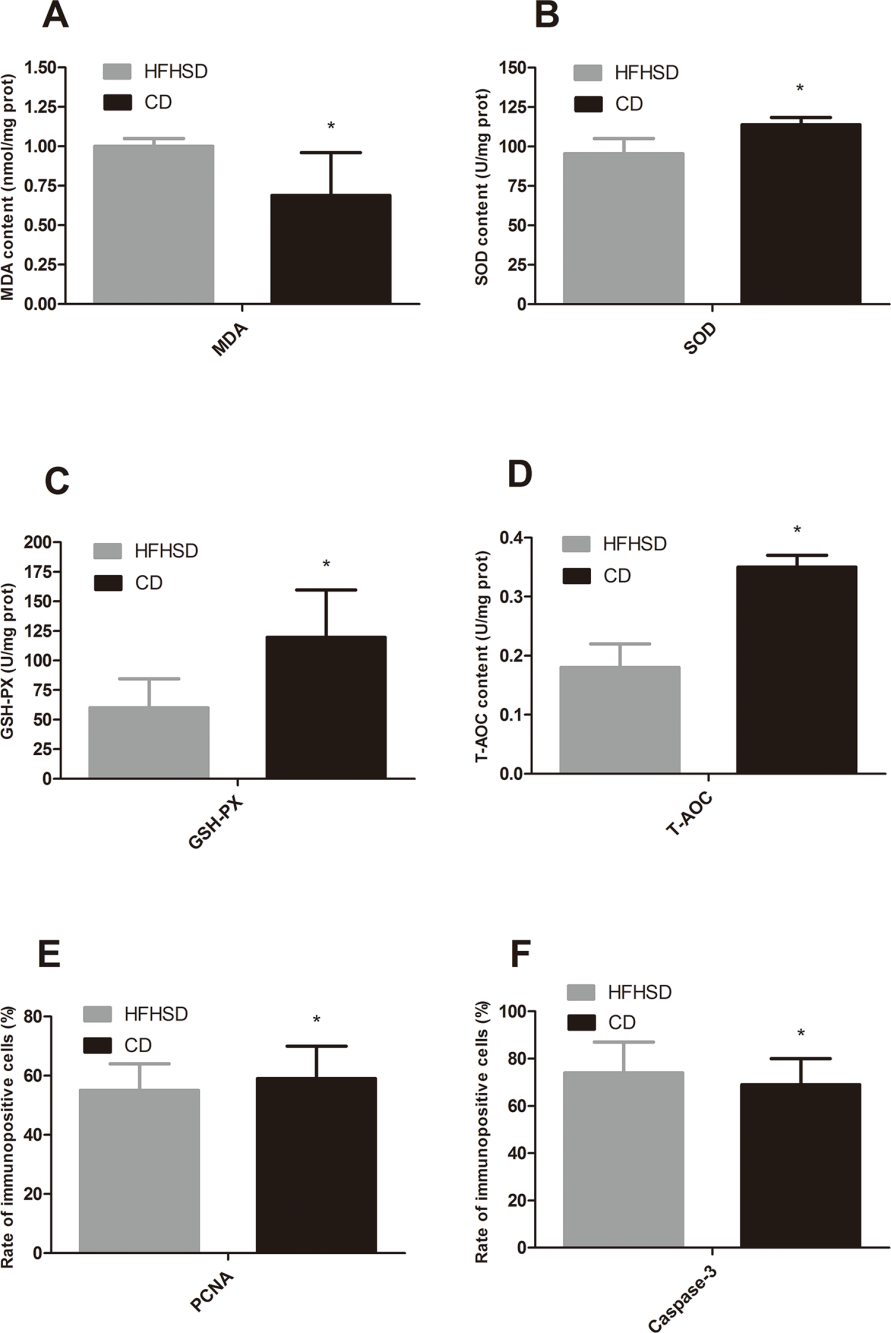
Changes in SOD, GSH-Px, T-AOC and MDA Levels in the Renal Tissue and Expression Levels of PCNA and Caspase-3 in the Renal Tubules in the HFHSD and CD groups. Notes: *P < 0.05 HFHSD vs. CD. CD = control diet, HFHSD = high-fat/high-sucrose diet.

Oil Red O staining revealed lipid deposition in the renal capsules and renal tubules in the minipigs in the HFHSD group but not in the CD group (Fig 4). The percentage of the positive area in the HFHSD group was 8.65% ± 9.58% compared with 0.17% ± 1.02% in the CD group (P = 0.11). These results indicated that the long-term HFHSD caused lipid aggregation in the kidney with subsequent lipid deposition. The expression levels of SREBP-1, FABP3, and LEPR were 2.67 times higher (P < 0.001), 1.32 times higher (P = 0.032), and 3.38 times higher, respectively, in the HFHSD than in the CD group (P = 0.001). The MDA level in the HFHSD group (1.00 nmol/mg protein) increased 44.37% compared with that in the CD group (0.69 nmol/mg protein) (*P* = 0.040) (Fig 5). The deposition of excessive amounts of lipids in the kidney results in lipotoxicity, which leads to inflammatory responses and oxidative stress [35, 36]. The expression levels of TNF-α, MIF, and IL-6 were increased 3.92 times (P = 0.039), 1.31 times (P = 0.019), and 1.31 times (P = 0.207) in the HFHSD compared with the CD group (Fig 2).

### Long-term HFHSDs decrease the antioxidant capacity of the kidney and promote apoptosis

The antioxidant system was impaired in the minipigs that received a high-fat/high-sucrose diet. The activity of SOD, which was 113.81 U/mg protein in the CD group, was reduced to 95.44 U/mg protein in the HFHSD group (a decrease of 16.14%, *P* = 0.032). The activity of GSH-Px, which was 119.51 U/mg protein in the CD group, was reduced to 60.12 U/mg protein in the HFHSD group (a decrease of 59.39%, *P* = 0.026). The levels of T-AOC were lower in the HFHSD group compared with the CD group (0.18 U/mg protein vs. 0.35 U/mg protein, respectively, a reduction of 47.36%, *P* = 0.045) (Fig 5).

**Fig 6:**
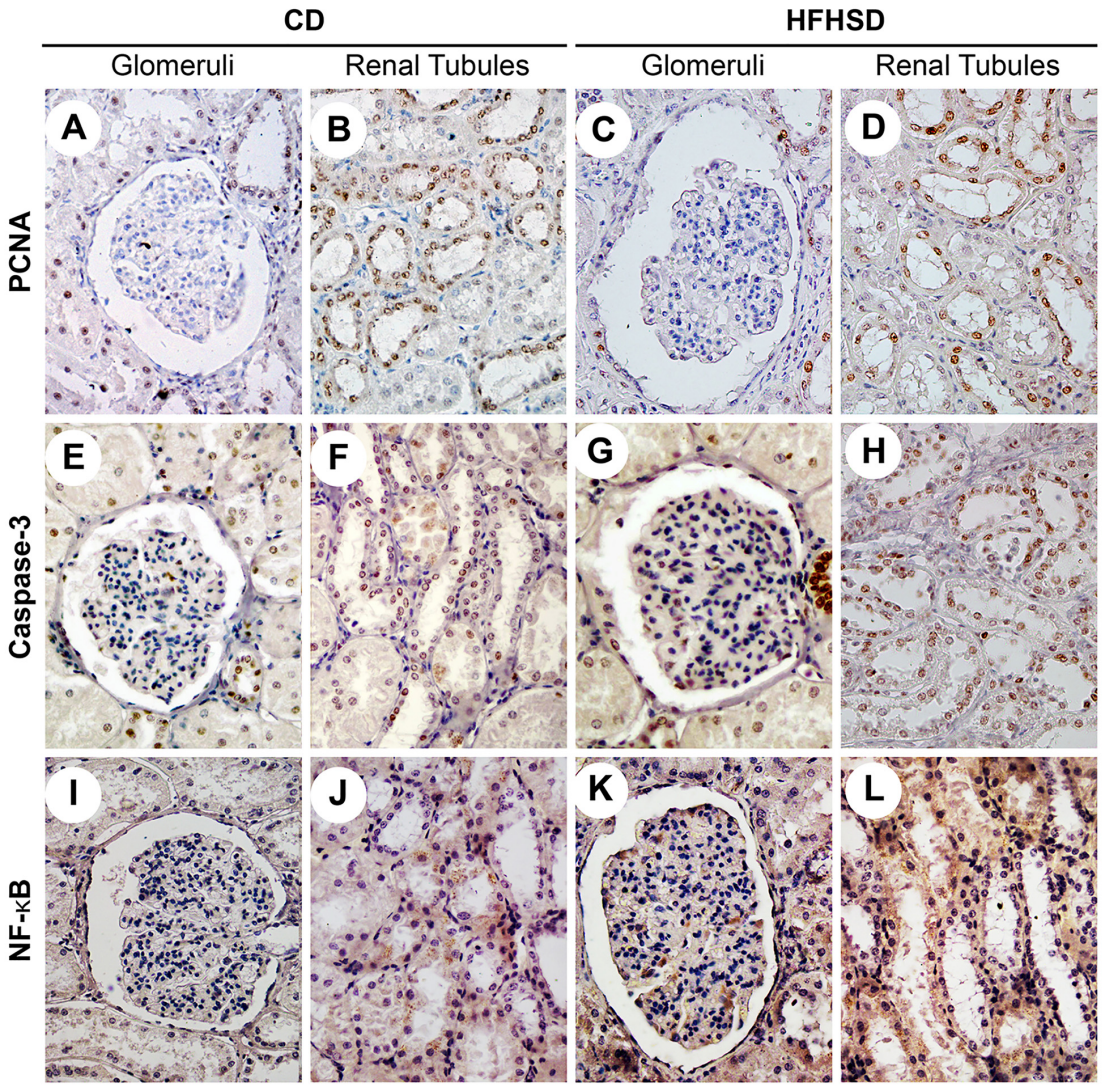
Comparison of PCNA, Caspase-3, and NF-κB in the Renal Tubules and Glomeruli in the HFHSD and CD Groups. A. A glomerulus in the CD group (PCNA). B. Renal tubules in the CD group (PCNA). C. A Glomerulus in the HFHSD group (PCNA). D. Renal tubules in the HFHSD group (PCNA). E. A glomerulus in the CD group (caspase-3). F. Renal tubules in the CD group (caspase-3). G. A glomerulus in the HFHSD group (caspase-3). H. Renal tubules in the HFHSD group (caspase-3). I. A glomerulus in the CD group (NF-κB). J. Renal tubules in the CD group (NF-κB). K. A glomerulus in the HFHSD group (NF-κB). L. Renal tubules in the HFHSD group (NF-κB). Bar = 50 μm. PCNA-positive cells were located mainly in the renal tubules. There were fewer PCNA-positive cells in the HFHSD group compared with the CD group. Caspase-3 was expressed primarily in the renal tubules. The number of caspase-3-positive cells in the HFHSD group was higher than the number of positive cells in the CD group. NF-κB was expressed primarily in the cytoplasm in the CD group; however, positive nuclear expression was also observed in the HFHSD group. CD = control diet, HFHSD = high-fat/high-sucrose diet.

The immunohistochemical staining showed positive PCNA expression (brown-yellow particles) localized in the nuclei of the proximal convoluted tubules and distal convoluted tubules. Positive caspase-3 expression (brown-yellow fine particles) was localized in the nuclei and cytoplasm of the distal convoluted tubules, with fewer positive cells in the glomeruli (Fig 6). PCNA expression in the renal tubules was reduced 5.87% (*P* = 0.042) in the HFHSD group compared with the CD group. Caspase-3 expression increased by 6.47% (*P* = 0.021) in the HFHSD group compared with the CD group (Fig 5). NF-κB was expressed primarily in the renal tubules in the CD group, especially in the distal convoluted tubules and the proximal convoluted tubules, and sometimes in cell nuclei. In the HFHSD group, NF-κB was expressed primarily in the cytoplasm and nuclei of the glomeruli and renal tubules (Fig 6).

## Discussion

With increases in food production capabilities, people are more likely to adopt high-fat/high-sugar diets that can lead to weight gain and obesity. Obesity is accompanied by the accumulation of fat, which can lead to chronic metabolic diseases such as metabolic syndrome that cause renal injury [37–39]. The majority of the previous studies investigating human renal disease have used rodents as a model organism; however, the observed pathological changes were not consistent with those observed in human renal diseases. Although the characteristics associated with metabolic syndrome, such as obesity, hypertension, and high blood glucose, were present [18, 40–42], all of these studies were one-sided. Some studies in minipigs also demonstrated corresponding renal injury [43]. In the present study, the minipigs developed metabolic syndrome phenotype after 23 months of a HFHSD. The renal histology showed glomerular swelling, mesangial matrix hyperplasia, widening of the renal capsular space, apoptosis of tubular epithelial cells, and lipid deposition in the renal tubules. The qRT-PCR results showed significantly different levels of the inflammation-associated genes TNF-α and MIF and the lipid deposition-associated genes SREBP-1, FABP3 and LEPR in the HFHSD compared with the CD group.

The minipigs in the HFHSD group had severe obesity after 23 months of induction. Oil Red O staining revealed large amounts of lipid deposition in the renal tubules. The HFHSD resulted in a rapid increase in body weight in the minipigs. In these minipigs, dyslipidemia increased the reabsorption of lipids by the renal tubules, resulting in a compensatory dilation of the blood vessels in the kidney and an increased glomerular filtration rate [44]—a major marker of early stage renal injury. Compared with the CD group, the unit area of the glomeruli in the HFHSD group decreased, which might have been associated with the swelling observed in the kidney and glomeruli. The glomus ratio decreased in the glomeruli, while that in the capsular space and the mesangial matrix increased in the HFHSD group compared with the CD group. Thus, the glomerular filtration rate changed, allowing proteins to pass more easily through the filtration system. We speculated that these changes were associated with proteinuria, which eventually decreases renal function. Changes in the glomerular structure prior to proteinuria can be detected, for example, based on the thickening of the glomerular basement membrane (GBM) [45]. PCNA is present in proliferating cells, and VEGFA is mainly associated with vascular endothelial cell proliferation and differentiation. Both proteins are both involved in the thickening of the capsular basement membrane. We detected PCNA and VEGFA by qRT-PCR, using samples (including cortex and medulla) that consisted of a mixture of glomerular, capsular and tubule cells. Thus, the data included changes in glomerular, capsular and tubule components. Based on the immunohistochemistry data, PCNA was mainly expressed in the renal tubule cells and decreased in the HFHSD group (0.57 ± 0.10) compared with the CD group (0.79 ± 0.23). Based on the HE staining, there was an increase in the renal capsular space. VEGFA expression was 1.91 ± 0.32 in the HFHSD group compared with 1.63 ± 0.46 in the CD group (P = 0.25).

Cellular apoptosis occurs extensively during normal tissue developmental to remove excessively proliferating cells and improve or delay renal interstitial fibrosis. However, if cellular apoptosis is overly active, the number of glomerular-intrinsic cells will be greatly reduced, resulting in the development of glomerulosclerosis [46]. During renal injury in rats, the glomeruli and renal tubular cells undergo excessive apoptosis, leading to the development and progression of renal disease [47]. In human kidney disease, renal tubular cells undergo significant apoptosis [48]. In this study, a high-fat/high-sucrose diet induced significant apoptosis in the renal tubular cells and caused renal tubular cell injury, while the glomerular cells did not undergo significant apoptosis. These results suggest that during the early stage of renal disease, renal tubular cell injury precedes glomerular cell injury. Renal tubular cell apoptosis might cause renal tubular atrophy and chronic renal insufficiency, eventually resulting in renal tissue injury. Caspase-3 is an important molecule that participates in apoptotic signal transduction. Caspase family members are key downstream enzymes in cellular apoptosis, among which caspase-3 is the central regulator and effector molecule of apoptosis [49]. The expression of caspase-3 in the CD group suggested that it plays an important role in the maintenance of cell growth in renal tissues and the clearance of abnormal cells. It is also an important regulatory molecule in the process of normal cell growth and has important functions in renal tissue homeostasis. Nuclear transcription factor NF-κB is a key factor in chronic inflammation. When activated, it induces the expression of many other pro-inflammatory cytokines, as well as a variety of inflammatory response-related cytokines that induce tissue inflammatory responses. The development of renal diseases caused by obesity from HFHSDs is associated with NF-κB activation. NF-κB activation induces the expression of pro-inflammatory cytokines and cytokine genes associated with inflammatory responses such as TNF-α and IL-1. These inflammatory factors cause renal tissue injury by reducing blood flow and filtration rates and modifying barrier functions in the capillary wall. NF-κB can regulate the transcription of inflammatory factors in mesangial and tubular epithelial cells; therefore, it plays a central role in the development and progression of renal disease [50]. The present study showed that in HFHSD-fed Bama minipigs, NF-κB was activated in the renal tubules and translocated to the nucleus, where it likely activated inflammatory factors to induce cellular apoptosis. The activation of NF-κB indicated that during the early stage of disease development, the inflammatory responses might be responsible for the induction of apoptosis.

Recently, it has been shown that oxidative stress plays a crucial role in the development and progression of glomerular injury caused by hyperlipidemia [51]. The antioxidant levels and activity and lipid peroxidation levels in the renal tissues of Bama minipigs in the HFHSD group revealed a decrease in SOD activity and a significant increase in MDA levels, which is consistent with the oxidative stress that occurs in human renal diseases. Oxidative stress can promote the progression of renal tissue disease. Excessive oxidative stress occurs in the renal tissues of diabetic rats, and the imbalance between antioxidation and oxidation aggravates the progression of renal diseases [52]. Streptozotocin-induced diabetic rats display oxidative stress in their renal tissues, which is accompanied by obvious glomerulosclerosis and tubulointerstitial fibrosis. In that study, antioxidant treatment decreased oxidative stress and relieved tubulointerstitial fibrosis and proteinuria [53]. MDA, the end product of oxidation, plays a fundamental role in lipid peroxidation, which results in the aggregation of macromolecules such as proteins and nucleic acids and subsequent cytotoxicity. The antioxidant system (e.g., SOD, catalase (CAT), and GSH-Px) plays an important role in the protection of normal renal tissue functions. GSH-Px can reduce lipid peroxidation induced by reactive oxygen species, clear harmful products, and protect the integrity of cell membranes. Harmful superoxide radicals can be transformed into hydrogen peroxide by SOD. Hydrogen peroxide is further decomposed into oxygen and water by CAT and GSH-Px. A variety of antioxidants function synergistically to remove excessive free radicals in the body and thus maintain homeostasis. The stability of tissues requires the maintenance of the relative balance between oxidants and antioxidants. When this balance is abrogated, oxidative stress can result in tissue damage. The antioxidant capacity in the Bama minipigs that were fed a HFHSD decreased significantly compared to that in the minipigs that received the CD, while lipid peroxidation resulted in an increase in oxidative stress. Thus, we postulate that oxidative stress was the main cause of the apoptosis observed in the renal tubule.

The present findings showed that long-term consumption of HFHSDs results in obesity and renal injury in Bama minipigs, providing a basis for further studies investigating renal tissue damage in metabolic syndrome.
